# Delayed Diagnosis of Primary Hyperoxaluria and Systemic Oxalosis in a Hemodialysis Patient: A Case Report and Literature Review

**DOI:** 10.7759/cureus.104479

**Published:** 2026-03-01

**Authors:** Hanane Benali, Mehdi El Mansouri, Ismail Ait Elkihal, Nabil Hamouche, Mariam Chettati, Wafaa Fadili, Inass Laouad

**Affiliations:** 1 Nephrology, Centre Hospitalo-Universitaire Mohammed VI de Marrakech, Marrakech, MAR; 2 Faculty of Medicine and Pharmacy Marrakech, Cadi Ayyad University, Marrakech, MAR

**Keywords:** anemia, bone deformities, end-stage chronic kidney disease, hyperoxaluria, pruritus

## Abstract

Primary hyperoxaluria is a rare congenital metabolic disorder characterized by excess production and accumulation of oxalate due to a hepatic enzyme deficiency. We report a rare case of a 54-year-old patient on chronic hemodialysis with initial nephropathy of lithiasic uropathy, in whom metabolic evaluation had not been performed. After six years of hemodialysis, the patient developed severe chronic pruritus, a tumoral syndrome with hepatosplenomegaly, peripheral lymphadenopathy, numerous subcutaneous masses, and diffuse bone pain. Laboratory tests revealed a profound anemia resistant to erythropoietin, severe hypoparathyroidism, and diffuse osteolytic lesions in the spine and pelvis.

CT-guided biopsy of the bone lesions showed deposits of calcium oxalates. The patient's condition deteriorated with the onset of central and peripheral neurological disorders, general deterioration, and eventual death from septic shock originating from a pulmonary focus.

## Introduction

Primary hyperoxaluria (PH) is a group of rare autosomal recessive metabolic disorders caused by specific enzymatic defects in the hepatic glyoxylate metabolism, leading to excessive endogenous oxalate production and subsequent calcium oxalate deposition in the kidneys and other tissues. Three main subtypes of PH have been identified based on the underlying genetic mutations: PH type 1 (PH1) due to alanine-glyoxylate aminotransferase (AGXT) deficiency, PH type 2 (PH2) resulting from glyoxylate reductase/hydroxypyruvate reductase (GRHPR) deficiency, and PH type 3 (PH3) caused by mutations in the HOGA1 gene encoding 4-hydroxy-2-oxoglutarate aldolase [1].

Clinically, PH is characterized by recurrent urolithiasis, progressive nephrocalcinosis, and gradual deterioration of renal function leading to end-stage renal disease (ESRD). Once renal failure occurs, systemic oxalosis develops due to widespread deposition of calcium oxalate crystals in bones, retina, myocardium, and other organs. These complications contribute to significant morbidity and mortality, especially when diagnosis is delayed.

Diagnosis is frequently established at an advanced stage because early symptoms are nonspecific and may mimic more common causes of nephrolithiasis in childhood. Timely recognition of the disease through biochemical and genetic testing is essential, as novel therapeutic approaches, such as RNA interference therapy targeting hepatic oxalate production, have shown promise in improving patient outcomes [2].

Given the severe clinical course and the need for early diagnosis and intervention, understanding the epidemiological, biochemical, and genetic characteristics of PH remains a crucial step toward optimizing patient management and survival.

## Case presentation

We report a rare case of a 54-year-old male patient referred from a private hemodialysis center for evaluation of bone deformities and erythropoietin-resistant anemia.

The patient had a history of renal lithiasis (a single calculus) diagnosed eight years earlier, which remained untreated and without etiological investigation. He was diagnosed with end-stage chronic kidney disease in 2018 and started on hemodialysis due to unexplored lithiasic uropathy. The disease course was marked by the development of erythropoietin-resistant anemia, for which he required multiple blood transfusions.

Eight months before admission, the patient developed progressively appearing, hard, painless subcutaneous axial masses associated with localized pruritus, evolving in a context of general health deterioration. Two months later, he was hospitalized in the internal medicine department for suspected histiocytosis (biopsy findings revealed histiocytic features under light microscopy, but the diagnosis was excluded due to the absence of anti-CD1a and anti-S100 antibodies). He was subsequently referred to our department for further evaluation and management.

On admission, the patient was conscious but appeared chronically ill, pale, and afebrile, with a heart rate of 89 beats per minute and blood pressure of 120/60 mmHg. There was no lower limb edema, and pulmonary auscultation revealed bilateral basal crackles. He reported intense pruritus refractory to treatment.

**Figure 1:**
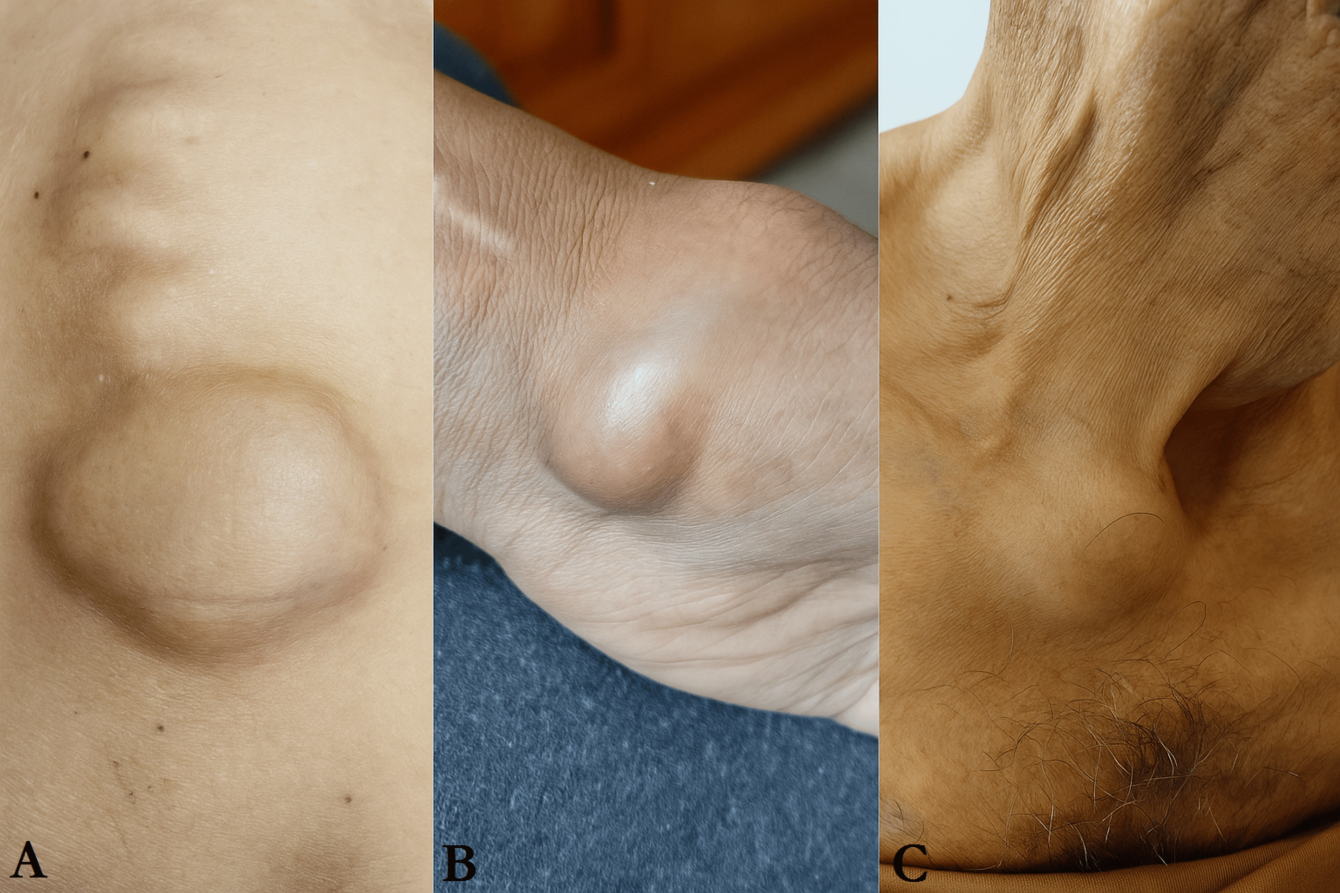
Bone deformities involving the axial skeleton (A), carpal region (B), and clavicle (C).

Osteoarticular examination revealed bone deformities involving the axial skeleton, clavicle, and carpal region (Figure 1), with tenderness on palpation. Pulmonary examination demonstrated bilateral pleural effusion. Abdominal examination revealed homogeneous hepatosplenomegaly and a firm, mobile 1-cm left axillary lymph node. Cardiovascular examination was unremarkable.

Lumbar CT scan revealed multilevel vertebral compression associated with diffuse osteolytic lesions throughout the spine, a pseudo-tumoral calcinosis adjacent to the L2 spinous process, and multilevel global disc protrusions from L3-L4 to L5-S1, appearing to impinge bilaterally on the L4 nerve roots at the L4-L5 level.

**Figure 2:**
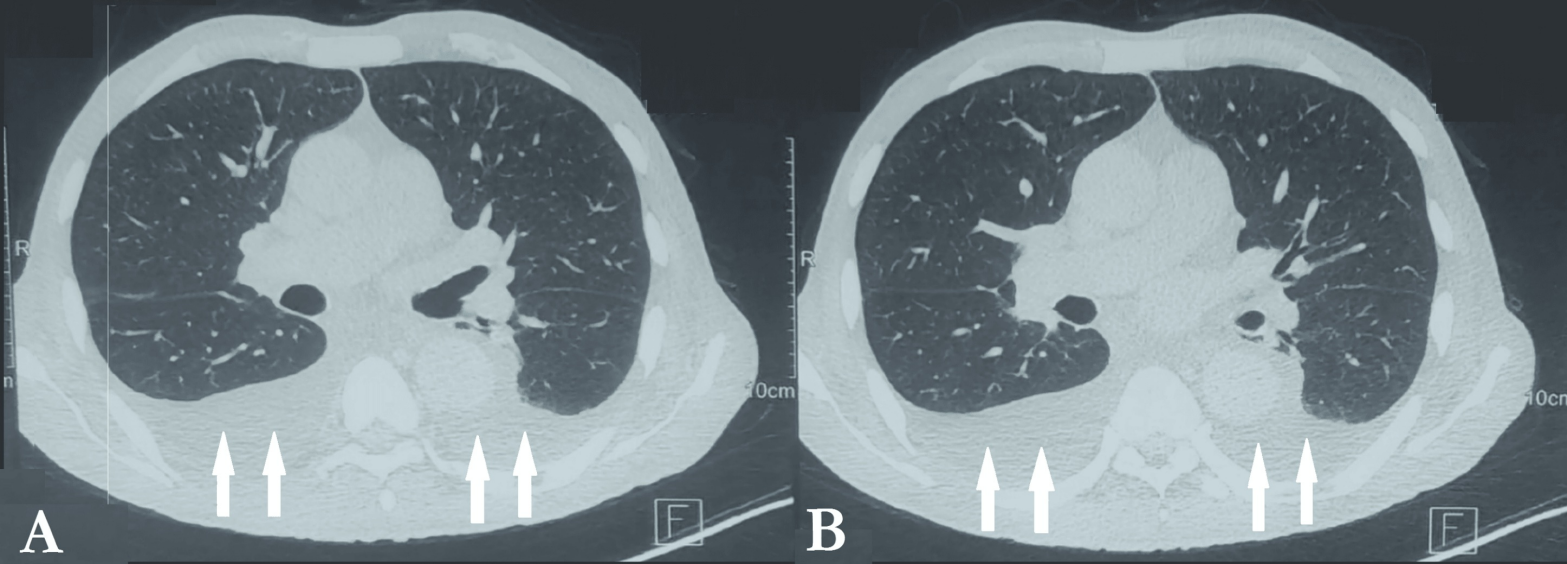
Thoraco-abdominal CT scans demonstrating moderate bilateral pleural effusion. (A) Bilateral pleural effusion. (B) Bilateral pleural effusion.

**Figure 3:**
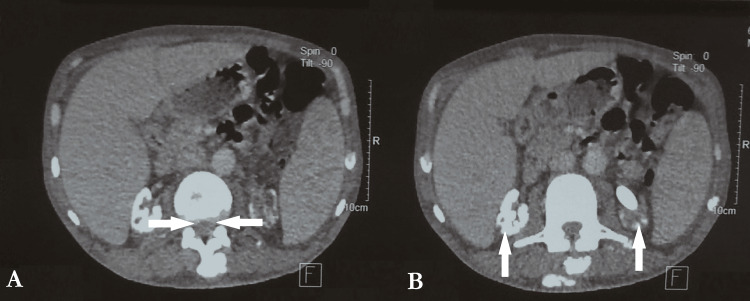
Abdominopelvic CT scans revealing lumbar vertebral compression and nephrocalcinosis. (A) Lumbar vertebral compression. (B) Nephrocalcinosis.

Thoraco-abdominopelvic CT (CT-TAP) showed cortical nephrocalcinosis, homogeneous hepatosplenomegaly, moderate bilateral pleural effusion and ascites (Figure 2), diffuse bone and periarticular soft tissue involvement, and kidneys with features of chronic nephropathy containing calcifications, including a large non-obstructive calculus in the lower calyceal group of the left kidney (Figure 3).

Laboratory tests (Table 1) revealed normochromic normocytic anemia with a hemoglobin level of 6.8 g/dL. The peripheral blood smear showed rouleaux formation without atypical immature cells. Bone marrow aspiration demonstrated 3% plasma cells. Serum ferritin was elevated at 340 µg/L.

**Table 1:** Summary of laboratory findings The symbol ♂ indicates male sex.

Parameter	Result	Reference Range
Hemoglobin	6.8 g/dL	13–17 g/dL (♂)
C-reactive protein	50 mg/L	<5 mg/L
Serum calcium	99 mg/L (2.47 mmol/L)	85–105 mg/L (2.1–2.6 mmol/L)
Serum phosphate	109 mg/L (3.52 mmol/L)	25–45 mg/L (0.8–1.45 mmol/L)
Parathyroid hormone	11 pg/mL	10–65 pg/mL
Alkaline phosphatase	139 IU/L	40–150 IU/L
Vitamin D (25-OH)	31 ng/mL	30–100 ng/mL
Aspartate aminotransferase	5 IU/L	<35 IU/L
Alanine aminotransferase	16 IU/L	<45 IU/L
Gamma-glutamyl transferase	109 IU/L	<60 IU/L
Plasma oxalate (pre-dialysis)	30.6 µmol/L	<40 µmol/L
Plasma oxalate (post-dialysis)	33 µmol/L	<40 µmol/L

The anemia was resistant to erythropoietin therapy (250 IU/kg/week), necessitating multiple blood transfusions. There was a significant inflammatory syndrome, with an erythrocyte sedimentation rate (ESR) of 120 mm/h and C-reactive protein (CRP) persistently elevated at 50 mg/L. Serum free light chain assay revealed increased levels of kappa and lambda chains (105.54 mg/L and 152.9 mg/L, respectively). Serum protein electrophoresis demonstrated moderate polyclonal hypogammaglobulinemia associated with marked hypoalbuminemia and a strong inflammatory response.

Calcium-phosphate metabolism evaluation showed normal serum calcium (99 mg/L), hyperphosphatemia (109 mg/L), and normal parathyroid hormone (PTH) level (11 pg/mL). Alkaline phosphatase was 139 IU/L, and vitamin D was 31 ng/mL. Liver function tests revealed no hepatic cytolysis (aspartate aminotransferase 5 IU/L, alanine aminotransferase 16 IU/L, gamma-glutamyl transferase 109 IU/L). Plasma oxalate levels were within normal limits (30.6 µmol/L) and remained stable after hemodialysis (33 µmol/L).

A salivary gland biopsy (BGSA) was also performed and returned normal results.

**Figure 4:**
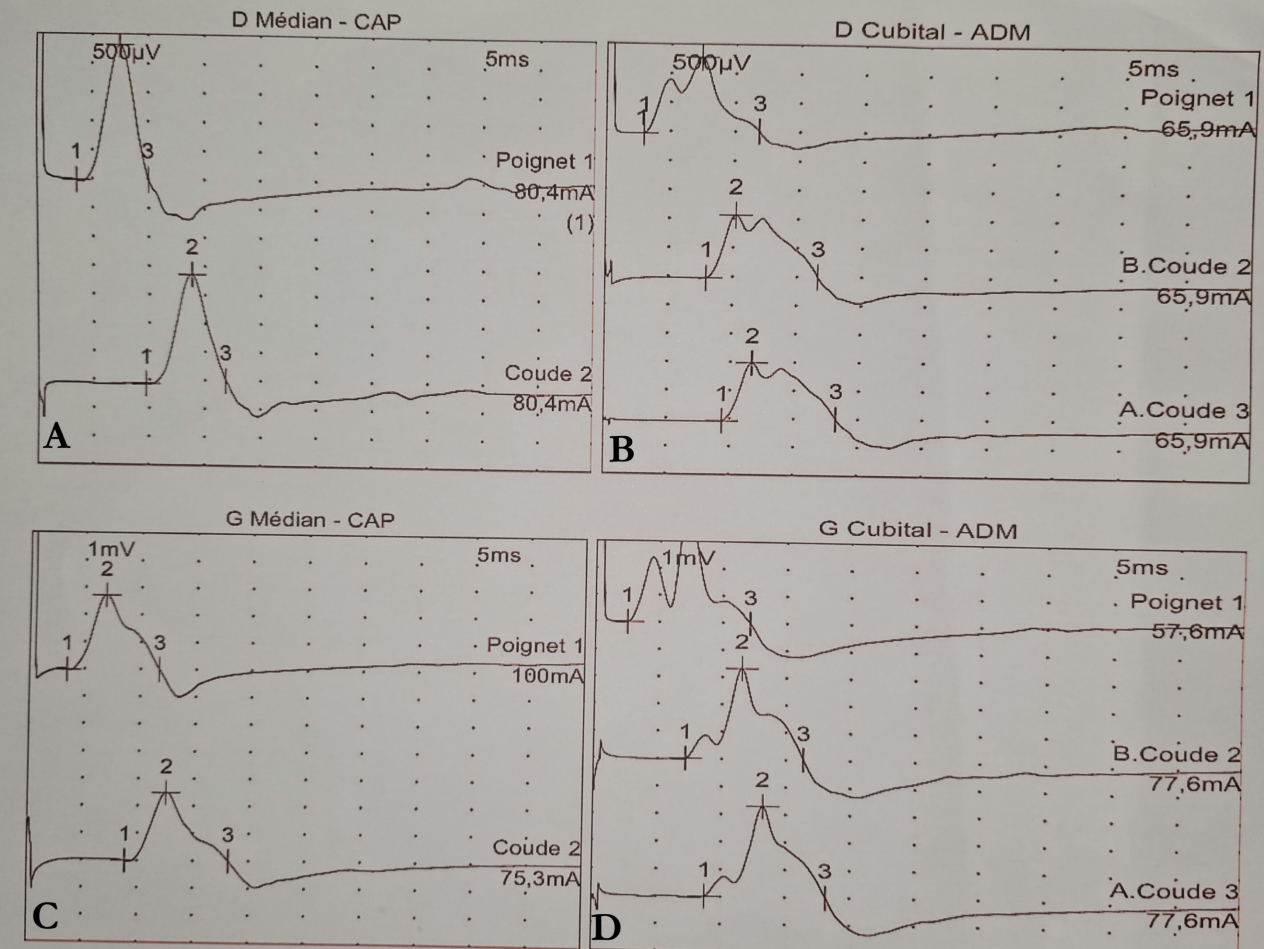
EMG revealing axonal involvement of both upper limbs. (A) Distal median-compound action potential. (B) Distal ulnar-abductor digiti minimi. (C) Proximal median-compound action potential. (D) Proximal ulnar-abductor digiti minimi.

The patient subsequently developed cognitive impairment with confusion and memory disturbances. Electromyography (EMG) revealed peripheral axonal neuropathy involving all four limbs (Figure 4).

A bone biopsy was performed at the level of the iliac bone. Macroscopic examination showed a firm, beige biopsy core measuring 1.3 cm in length and entirely embedded in a single cassette. Histological examination showed fibrous, striated muscle, and osseous tissue extensively remodeled by fibrosis and hemorrhagic suffusions. There was a prominent granulomatous inflammatory resorptive reaction composed mainly of foreign-body type giant cells surrounding crystalline deposits. These deposits were frequently arranged in rosette-like structures, appearing optically clear on hematoxylin-eosin staining and birefringent under polarized light. A few bony sequestra were noted at the periphery. No malignant tumor proliferation was identified.

Spectrophotometric analysis performed on the previously retained calculus (passed eight years earlier) confirmed that it was composed of calcium oxalate.

## Discussion

PH is a congenital hepatic metabolic disorder that may be hereditary or, in rare cases, acquired. It results from excessive endogenous oxalate production, in contrast to secondary hyperoxaluria, which arises from excessive dietary oxalate intake or increased intestinal absorption, leading to elevated urinary oxalate excretion [3-4].

To date, three distinct inherited enzymatic deficiencies have been identified as causes of PH1, PH2, and PH3, although emerging evidence suggests that additional, as yet unidentified, enzymatic defects may exist. Excessive oxalate production leads to recurrent urinary lithiasis and progressive nephrocalcinosis, the hallmark features of PH. As renal damage progresses, the glomerular filtration rate (GFR) declines, ultimately resulting in chronic kidney disease and ESRD. In PH1, the most severe form, systemic oxalosis can develop once renal excretory function is lost.

The clinical presentation in our patient was consistent with previous reports, including a history of renal lithiasis (a single large calculus) and nephrocalcinosis [5-7]. However, the age at presentation was atypical, as only one similar case of systemic oxalosis has been described in a 47-year-old patient who was diagnosed with PH after six years of hemodialysis [6].

In our case, the main presenting features were bone deformities and localized pruritus, associated with a tumor-like syndrome comprising hepatosplenomegaly, lymphadenopathy, and deterioration of general health. This prompted extensive investigations to exclude hematologic malignancy. Ultimately, bone biopsy confirmed the diagnosis of the underlying nephropathy after six years of hemodialysis, revealing oxalate crystal deposition within bone tissue associated with extensive fibrotic remodeling and a resorptive granulomatous inflammatory reaction.

Bone lesions in systemic oxalosis are caused by the inflammatory response induced by oxalate crystals, leading to bone resorption. Spontaneous fractures may occur in weakened areas [8], though none were observed in our patient. Radiologic features typical of oxalosis include dense metaphyseal bands and vertebral osteosclerosis. However, distinguishing oxalosis-related lesions from those secondary to hyperparathyroidism in patients with ESRD is often challenging. Interestingly, our patient presented with hypoparathyroidism (6.6 pg/mL, measured eight months before admission), which may have exacerbated the bone lesions. The observed spontaneous normocalcemia could be related to osteoclastic activation mediated by macrophage-driven granulomatous inflammation. A similar case of systemic oxalosis associated with hypoparathyroidism and normocalcemia was reported in an 18-year-old patient in our department in 2014 [9].

From a therapeutic standpoint, combined liver-kidney transplantation remains the treatment of choice, especially in patients with ESRD [10]. This approach addresses both the metabolic defect (hepatic enzyme deficiency) and renal failure. However, this option was not feasible in our setting, and intensified daily dialysis was proposed instead.

## Conclusions

PH is a rare but potentially devastating inherited metabolic disorder, particularly when diagnosis is delayed until progression to ESRD. Late-onset presentations, such as in the present case, are exceptional and frequently characterized by atypical clinical and radiological features that may mimic hematologic malignancies or metabolic bone disorders, thereby posing significant diagnostic challenges. The association of skeletal deformities, hepatosplenomegaly, and lymphadenopathy reflects the systemic dissemination of oxalate deposits that occurs once renal excretory capacity is compromised.

From a therapeutic perspective, combined liver-kidney transplantation remains the only curative treatment for PH1, as it addresses both the underlying hepatic enzymatic defect and renal failure. Nevertheless, limited availability of transplantation, particularly in resource-constrained settings, highlights the critical importance of early diagnosis and timely intervention. Emerging therapeutic strategies, including intensified dialysis regimens and novel RNA interference-based therapies (such as lumasiran and nedosiran) targeting hepatic oxalate overproduction, represent promising alternatives that may improve outcomes and delay disease progression in selected patients.
